# Risk factors of internal carotid artery stenosis in patients with proliferative diabetic retinopathy: an analysis using optical coherence tomography and optical coherence tomography angiography

**DOI:** 10.1186/s12886-024-03391-z

**Published:** 2024-04-09

**Authors:** Chae Yoon Lee, Jung Min Park, Myeong In Yeom

**Affiliations:** Department of Ophthalmology, Maryknoll Hospital, 121 Junggu-ro, Jung-gu, 48972 Busan, Korea

**Keywords:** Internal carotid artery stenosis, Optical coherence tomography, Optical coherence tomography angiography, Carotid duplex ultrasonography, Proliferative diabetic retinopathy, Ocular ischemic syndrome

## Abstract

**Background:**

This research investigates the correlation between the severity of internal carotid artery (ICA) stenosis and retinal parameters in patients with proliferative diabetic retinopathy (PDR), aiming to uncover potential risk factors.

**Methods:**

A retrospective analysis of 68 patients (136 eyes) diagnosed with bilateral PDR from January 1, 2017, to December 31, 2021, was conducted. Carotid artery stenosis (CAS) was assessed using neck computed tomography angiography (CTA) and carotid duplex ultrasound (CDUS), with stenosis classified into two groups: normal (group 1) and mild or above (group 2), based on the North American Symptomatic Carotid Endarterectomy Trial (NASCET) criteria. Optical coherence tomography (OCT) and OCT angiography (OCTA) measured several retinal parameters, including sub foveal choroidal thickness (SFCT), retinal nerve fiber layer (RNFL) thickness, ganglion cell-inner plexiform layer (GCIPL) thickness, vessel density (VD), and foveal avascular zone (FAZ) area. Statistical analyses determined correlations between ICA degrees and retinal parameters.

**Results:**

This study showed significant differences between groups in total VD, FAZ area, total RNFL thickness, and temporal RNFL thickness, indicating that patients with more severe ICA stenosis had noticeable retinal changes. Other parameters such as hyperlipidemia, total cholesterol levels, and intraocular pressure (IOP) also differed significantly, while no notable differences were observed in SFCT, central VD, average GCIPL, and superior, nasal, and inferior RNFL thickness.

**Conclusion:**

The study findings highlight retinal changes, such as an increased FAZ area, decreased total VD, and a total and thinner temporal RNFL, which suggest the need for carotid artery evaluation in patients. These findings have important clinical implications for the need for carotid work up in patients with PDR.

**Supplementary Information:**

The online version contains supplementary material available at 10.1186/s12886-024-03391-z.

## Background

Proliferative diabetic retinopathy (PDR) and internal carotid artery (ICA) stenosis are interconnected conditions with implications for severe vision loss. PDR, an advanced stage of diabetic retinopathy, involves microvascular dysfunction due to chronic hyperglycemia, leading to neovascular fronds and increased risk of complications. Atherosclerosis, accelerated by diabetes, contributes to arterial narrowing in both carotid and retinal vessels, exacerbating ischemic changes in the retina and advancing PDR progression (1–2).

The prevalence of PDR with concurrent ICA stenosis varies among populations. A study by Wong et al. reported a prevalence rate of 6.8% for patients with PDR and ICA stenosis in a Chinese population [2]. Additionally, the mortality rates associated with this combination of conditions are concerning. A study conducted by Liu et al. found that individuals with DM, PDR, and ICA stenosis had a significantly higher mortality rate compared to those without ICA stenosis [3]. Studies, including those by Katakami et al. and Sasaki et al., show a significant association between PDR and ICA stenosis, suggesting shared mechanisms of microvascular dysfunction and atherosclerosis (4–5). Further research is needed to elucidate the exact pathophysiological mechanisms and potential therapeutic implications of this correlation.

However, understanding the intricate interplay between PDR and ICA stenosis is essential due to their potential bidirectional influence on each other. While ICA stenosis may exacerbate the symptoms and progression of PDR by limiting the blood supply to the retina, severe PDR might also contribute to the development or worsening of ICA stenosis through shared pathophysiological mechanisms such as microvascular dysfunction and atherosclerosis. It is pertinent to consider that ICA stenosis may act both as a consequence and an exacerbating factor of PDR, highlighting the need for a comprehensive understanding and approach in managing patients with these concurrent conditions. Through our study, we aim to shed light on the nuanced relationship between these two serious complications of diabetes mellitus, providing clinicians with valuable information for risk assessment and therapeutic decision-making.

### Objectives of the study

Diabetic retinopathy, particularly PDR, is a well-recognized complication of DM. Recent studies have shed light on a possible association between PDR and ICA stenosis [6], emphasizing the multifaceted nature of DM complications. Despite the established literature on risk factors for ICA stenosis - encompassing aspects like gender, older age, duration of diabetes, hypertension, hyperlipidemia, LDL cholesterol levels, and elevated HbA1c - there remains a paucity of information on how these factors interplay with ophthalmological parameters like best corrected visual acuity (logMAR) and intraocular pressure (IOP) in the context of ICA stenosis.

This study ventures beyond the conventionally recognized risk factors for ICA stenosis. Our objective is to elucidate unidentified factors that may predispose patients, even those without traditional risk factors, to ICA stenosis. Harnessing the capabilities of Optical Coherence Tomography (OCT) and Optical Coherence Tomography Angiography (OCTA), we aim to provide a sophisticated imaging technique to assess the retina in unparalleled detail. This could facilitate a more granular and quantitative understanding of retinal alterations pertinent to ICA stenosis.

Distinct from extant research, our study pioneers the integration of high-resolution retinal imaging with the exploration of unrecognized risk predictors for ICA stenosis. By bridging ophthalmological and vascular realms, our findings aspire to shape more refined management strategies, potentially altering the trajectory of complications arising from the confluence of PDR and ICA stenosis. This paper endeavors to fill the knowledge gaps, offering a fresh perspective and thereby piquing interest in the evolving narrative of DM complications.

## Methods

### Participants

We retrospectively analyzed 68 patients with bilateral proliferative diabetic retinopathy diagnosed between 2017 and 2021, including 136 eyes with the same severity of disease. The presence and severity of carotid artery stenosis(CAS) were assessed through the use of cervical vascular computed tomography or carotid artery ultrasonography.

This study was approved by the Institutional Review Board of Maryknoll Hospital, Busan (No. 2023 − 322). All procedures were conducted in compliance with the principles of the Declaration of Helsinki. Informed consent for publishing this study is not applicable since it is a retrospective study. In accordance with the Preferred Reporting Items for Systematic Reviews and Meta-Analyses (PRISMA) guidelines, the present study followed a structured and transparent approach to selecting, analyzing, and reporting the relevant literature.

**Fig. 1:**
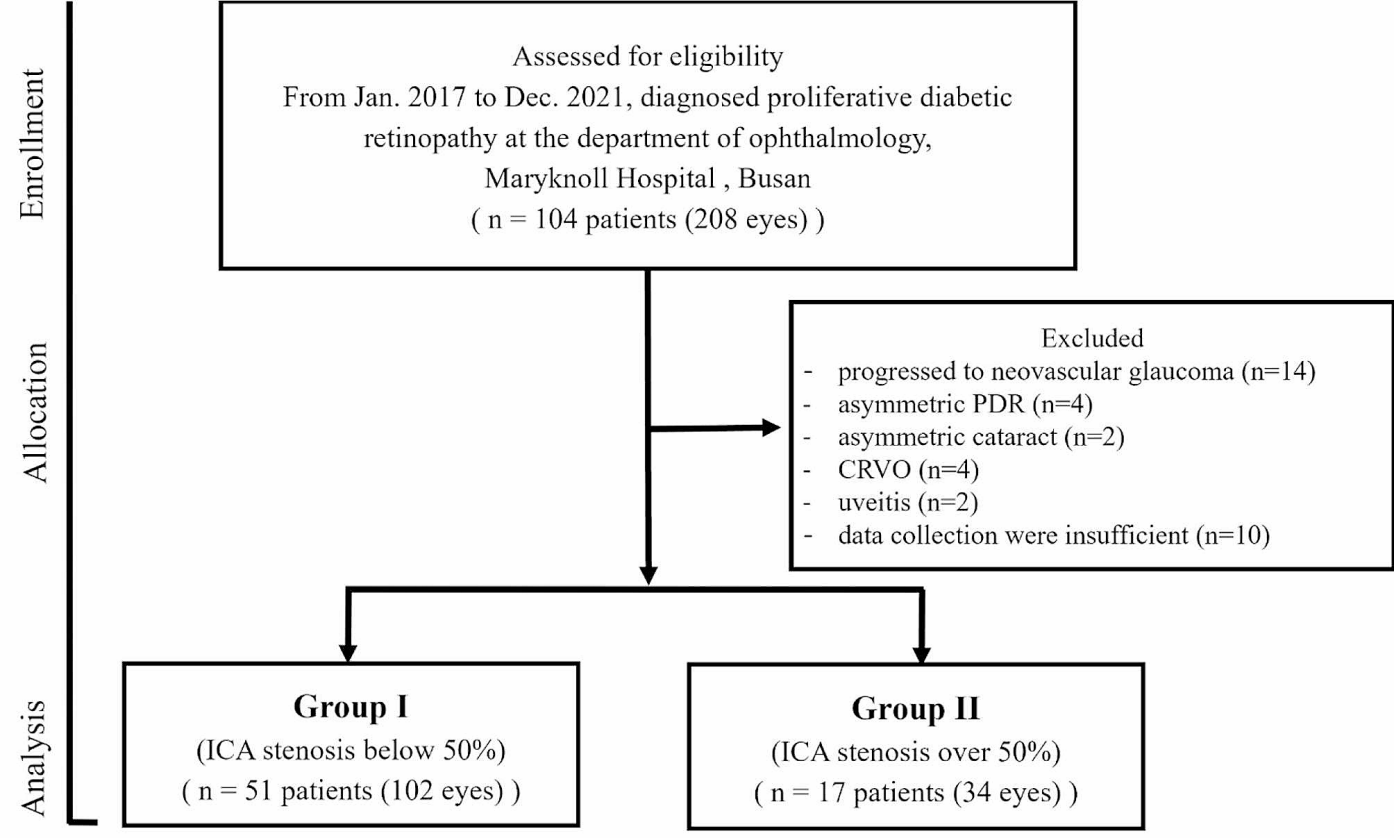
Patient’s enrollment

We excluded 36 patients from our study based on the following exclusion criteria: progression to neovascular glaucoma (*n* = 14), asymmetric proliferative diabetic retinopathy (*n* = 4), asymmetric cataract (*n* = 2), central retinal vein occlusion (*n* = 4), uveitis (*n* = 2), and insufficient data collection (*n* = 10). (Fig. 1)

### Trial design and study settings

We assessed the presence and severity of CAS using neck Computed Tomography Angiography (CTA) and Carotid Duplex Ultrasound (CDUS). The degree of ICA stenosis was initially classified into a four-grade system based on the North American Symptomatic Carotid Endarterectomy Trial (NASCET) criteria using neck CTA and a grayscale panel as determined by CDUS. The NASCET criteria is grounded in the measurement of the ICA lumen diameter at the maximal stenosis point and the diameter of the unaffected distal ICA. From these measurements, the degree of stenosis is calculated using a derived formula. The NASCET criteria broadly define three stenosis categories: less than 50%, 50–69%, and 70% or greater.

For the purpose of this study and to facilitate a focused analysis, we refined our approach by categorizing the degree of stenosis into two main groups:

Group 1: Represents patients with normal levels of stenosis (less than 50%).

Group 2: Includes patients with mild to severe levels of stenosis (50% and above).

To simplify the analysis, we categorized the degree of stenosis into two groups: group 1 corresponds to stenosis at the normal level (less than 50% stenosis), while group 2 includes stenosis at or above the mild level (more than 50% stenosis). Subsequently, OCT and OCTA (Cirrus HD OCT Model 5000, Carl Zeiss Meditec Inc., Dublin, CA, USA) were conducted to examine the various retinal parameters.

### Outcome assessments

To evaluate potential correlations between the degree of ICA stenosis and retinal parameters, we conducted a comparative analysis of main systemic factors for each group. We examined the subfoveal choroidal thickness(SFCT), changes retinal nerve fiber layer(RNFL) thickness, and average ganglion cell-inner plexiform layer(GCIPL) thickness by using OCT. Additionally, we evaluated superficial vascular density(SVD), and foveal avascular zone(FAZ) using OCTA. Statistical analysis was then performed to determine the correlation between the degree of CAS and retinal parameters.

### Statistical analysis

In this study, statistical analyses were carried out using IBM SPSS ver. 22.0 software (IBM Corp., Armonk, NY, USA). The paired t-test was used to examine the differences in clinical results and patient demographics between the two groups. An independent sample t-test was utilized to compare the measured values between the two groups based on the presence or absence of common carotid artery plaque for variables with a normal distribution. To confirm the correlation between the measured values and variables among groups based on the remaining ICA grades, a linear multivariate test was conducted using regression analysis. Power analysis was performed, and it revealed an effect size of 0.5, a statistical significance of 0.05, and a statistical power of 0.91 for both groups.

## Results

The study included subjects with an average age of 62.55 years and the demographic factors of two groups are shown in Table 1. The statistically significant differences in measured values between groups 1 and 2 were observed in total VD (13.73 ± 3.21, 11.53 ± 3.37, respectively; *p* = 0.003), FAZ area (0.53 ± 0.62, 0.56 ± 0.84, respectively; *p* = 0.002), total RNFL thickness (97.87 ± 5.542, 95.04 ± 4.10, respectively; *p* = 0.013) and temporal RNFL thickness (82.46 ± 27.74, 71.75 ± 20.18, respectively; *p* = 0.019), which showed significant thinning in group 2 with severe ICA stenosis. (Table 2.) However, no significant differences were observed in SFCT, central VD, average GCIPL and thickness of the superior, nasal, and inferior RNFL between group 1 and group 2. (Table 2.)

**Table 1:** The demographic factors of two groups

The demographic factors	Group 1	Group 2	*p*-value*
Sex (M/F)	35/16	10/7	0.321
Age (years)	61.5 ± 10.58	66.7 ± 7.52	0.003
Diabetes mellitus (years)	13.38 ± 9.23	16.46 ± 8.46	0.063
Hypertension (%)	46.70	62.50	0.375
Hyperlipidemia (%)	15.20	50	0.022
Total Cholesterol (mg/dL)	224.97 ± 91.71	164.41 ± 115.62	0.019
LDL Cholesterol (mg/dL)	91.89 ± 38.69	83.58 ± 41.49	0.191
HbA1c (%)	7.87 ± 1.33	8.16 ± 1.32	0.177
BCVA (log MAR)	0.41 ± 0.52	0.62 ± 0.70	0.089
IOP (mmHg)	15.78 ± 7.08	14.2 ± 2.62	0.042

Values are presented as mean ± SD unless otherwise indicated

BCVA = best corrected visual acuity; LogMAR = logarithm of the minimum angle resolution; IOP = intraocular pressure

**Table 2:** OCT and OCTA parameters of two groups

OCT and OCTA parameters	Group 1	Group 2	*p*-value*
SFCT (μm)	259.81 ± 46.66	253.12 ± 35.58	0.224
VD central (%)	2.83 ± 2.99	2.4 ± 2.26	0.359
VD total (%)	13.73 ± 3.21	11.53 ± 3.37	0.003
FAZ area (mm²)	0.53 ± 0.62	0.56 ± 0.84	0.002
average GCIPL (μm)	73.74 ± 26.39	70.25 ± 13.20	0.405
RNFL thickness (μm)			
Total	97.87 ± 5.542	95.04 ± 4.10	0.013
Superior	108.74 ± 34.48	104.62 ± 26.11	0.262
Inferior	111.21 ± 30.26	101.62 ± 27.23	0.07
Temporal	82.46 ± 27.74	71.75 ± 20.18	0.019
Nasal	73.68 ± 22.13	69.83 ± 19.30	0.202

Values are presented as mean ± SD unless otherwise indicated

SFCT = subfoveal choroidal thickness; VD = vessel density; FAZ = foveal avascular zone; GCIPL = ganglion cell layer inner plexiform layer; RNFL = retinal nerve fiber layer

**Fig. 2:**
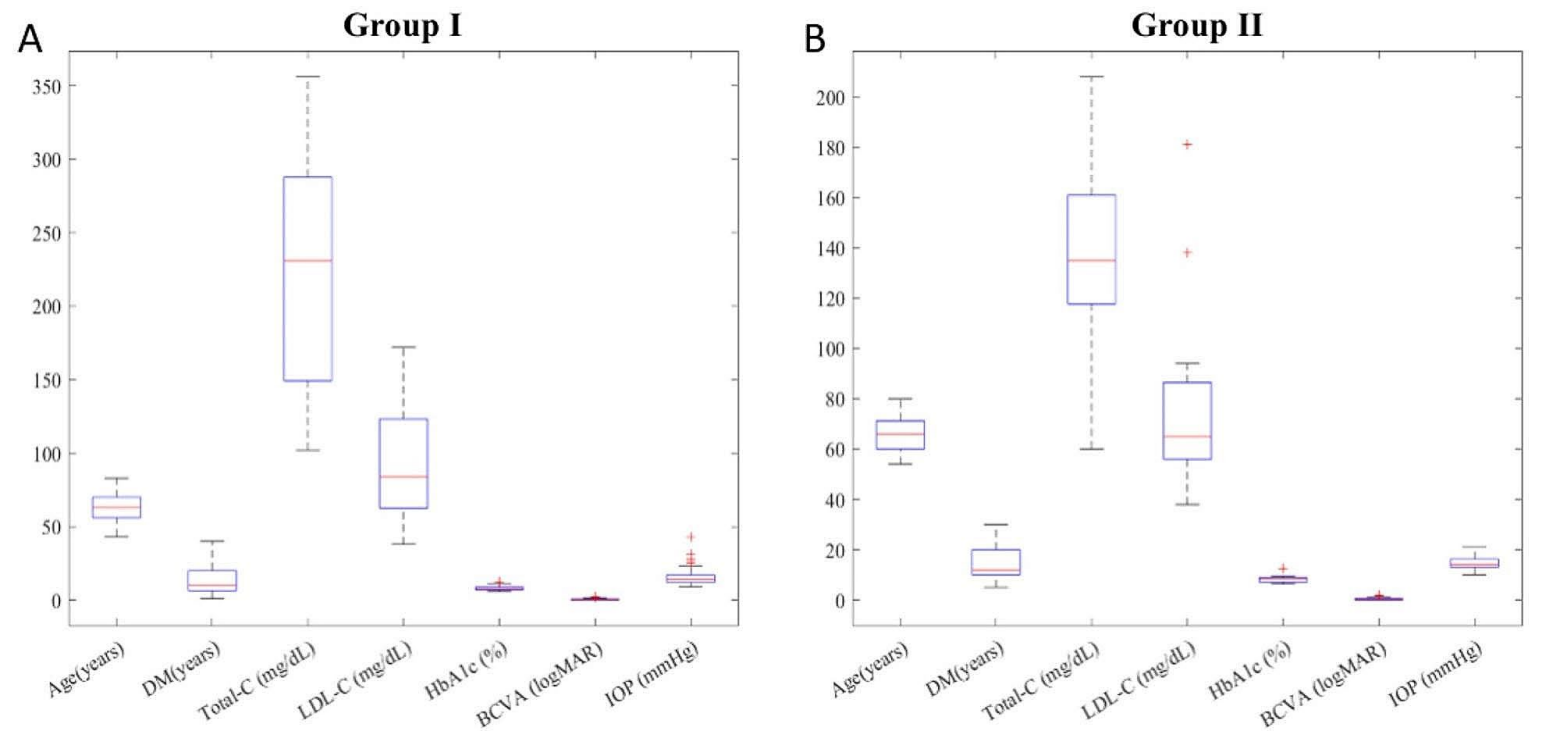
The box-plots of the systemic factors of group I and II

**Fig. 3:**
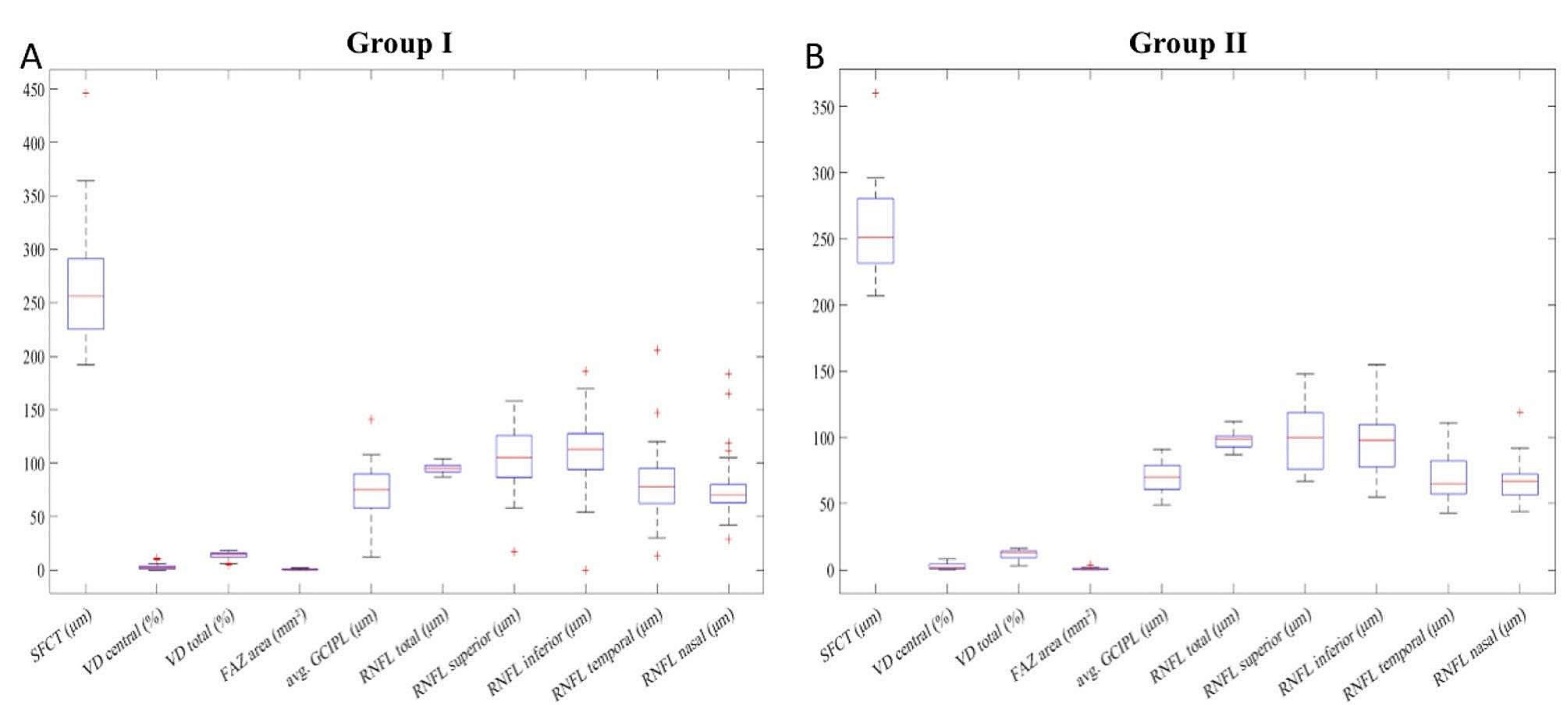
The box-plots of the OCT and OCTA parameters of group I and II

By examining the box plots of systemic factors for group I and group II in both Figs. 2 and 3, it becomes straightforward to identify the differences between the two groups.

**Fig. 4:**
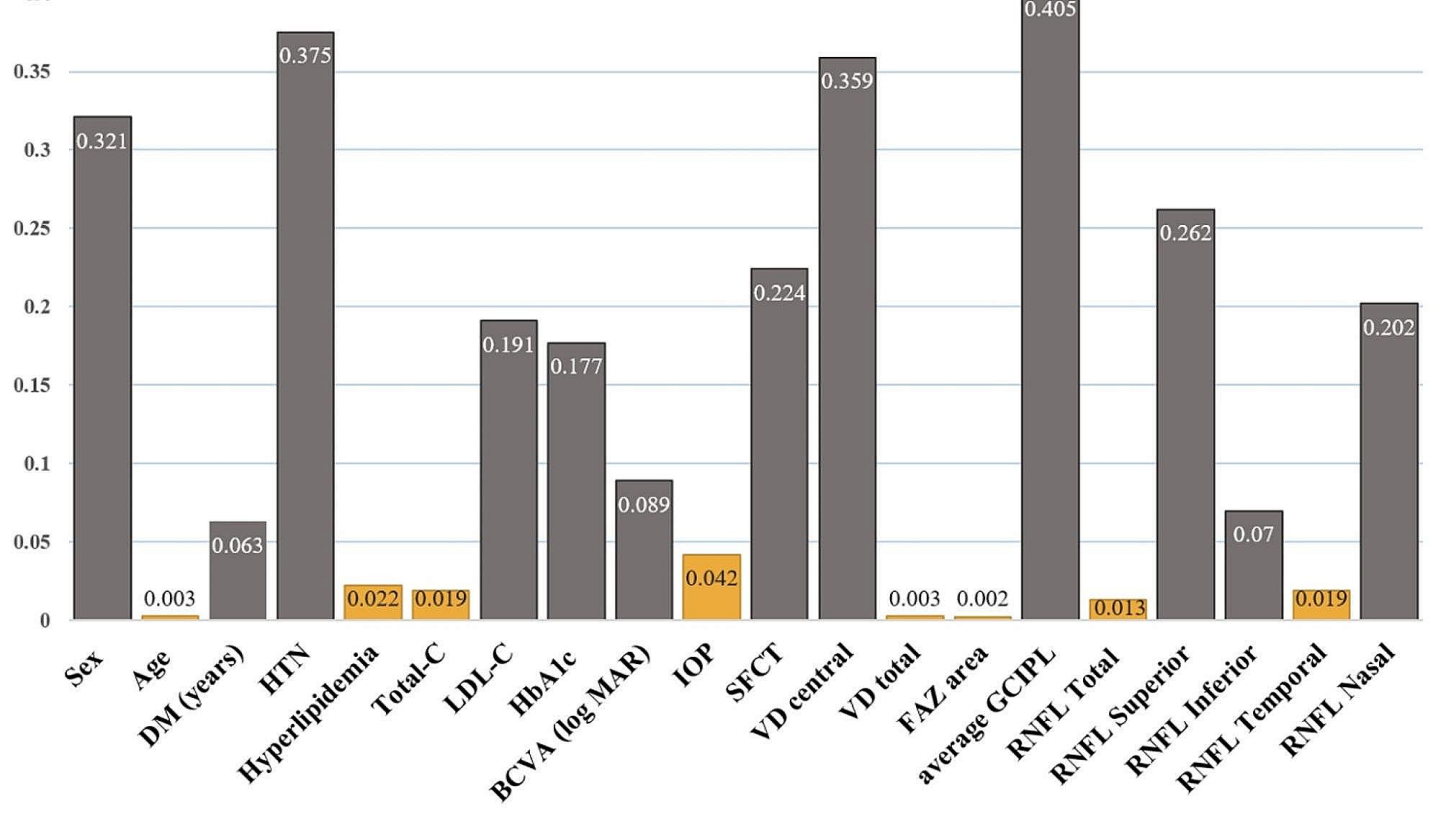
The histogram of *p*-values of the parameters between two groups

According to the results presented in Fig. 4, there were significant differences (*p*-value < 0.05) between two groups for several parameters, including total RNFL, temporal RNFL, FAZ area, total VD, age, hyperlipidemia, total cholesterol (total -C), and IOP, as indicated by the histogram of *p*-values.

## Discussion

### Decreased vascular density

Retinal vascular density is a quantitative measurement that describes the concentration or number of blood vessels within a specific area of the retina. It provides information about the health of the retinal vascular system. A higher VD indicates a greater number of blood vessels within the measured area, while a lower density suggests fewer vessels.

The study found that total vascular density decreased significantly in group 2 with severe ICA stenosis and longer duration of diabetes. Previous studies [7, 8] have suggested that severe ICA stenosis with DM can lead to reduced blood flow to the optic nerve head and retina, which may cause ischemia and subsequent vascular changes. Our findings support the existing evidence that suggests a correlation between ICA stenosis and vascular density in patients with proliferative diabetic retinopathy [7].

### Increased FAZ area

The foveal avascular zone is a small, circular area in the center of the retina known as the fovea. The FAZ is called avascular because it lacks the typical network of blood vessels that are present in other parts of the retina. In healthy eyes, the FAZ is relatively small and well-defined. However, certain eye conditions or disease can cause changes in the FAZ such may involve an enlargement or disruption of the FAZ area, which can affect central vision and lead to visual impairments.

The study observed a statistically significant increase in FAZ area among participants in group 2. The findings of this study are consistent with previous research suggesting that in patients with DM, the FAZ area increases with severe ICA stenosis [8, 9]. This could be attributed to reduced blood flow to the retina leading to ischemia, as suggested by Conrath J [10]. Additionally, Kikushima W. proposed that the size of the FAZ area could serve as a marker for the degree of ICA stenosis [11].

### Decreased total RNFL thickness / temporal RNFL thickness

**Fig. 5:**
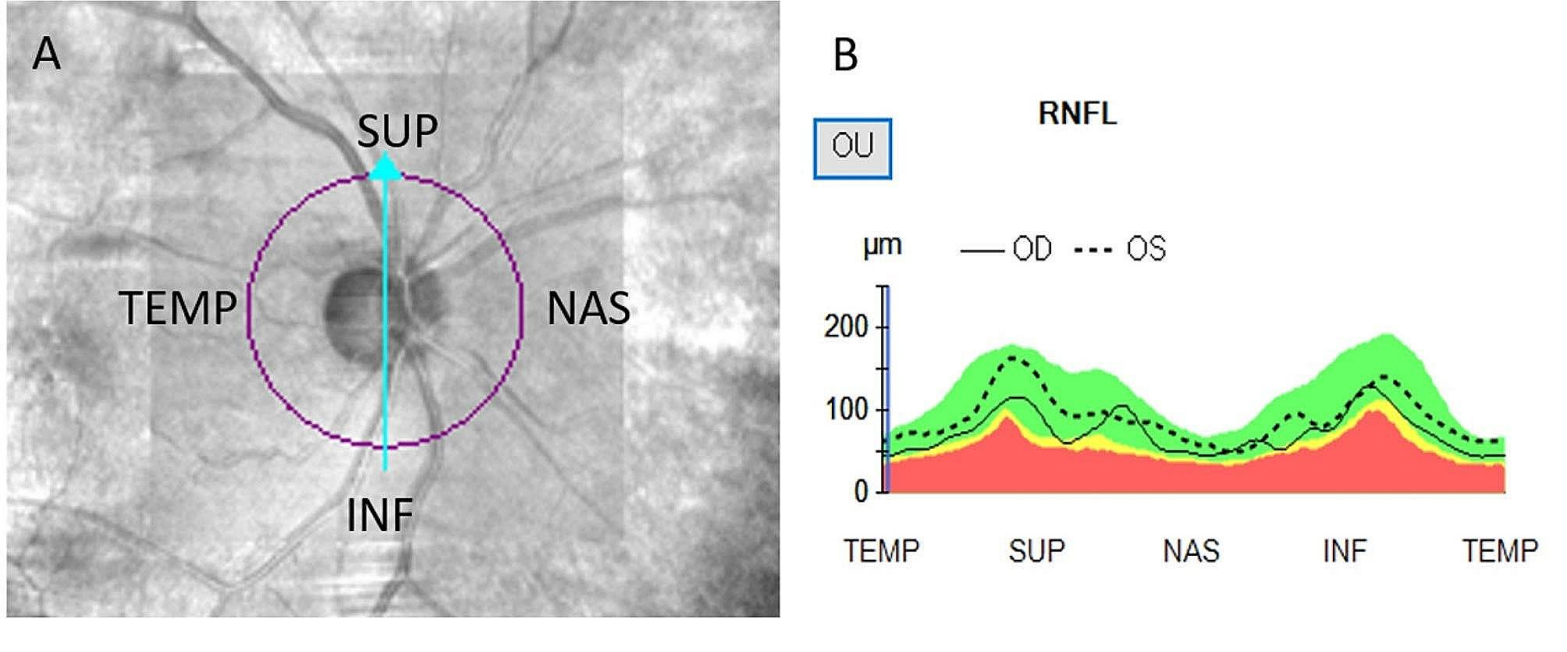
Report sample of the measurement of the retinal nerve fiber layer (RNFL) with OCT. (**A**) The four main regions of the RNFL. (**B**) RNFL thickness of four regions

The retinal nerve fiber layer is a thin layer of nerve fibers that lines the inner surface of the retina, which is the light-sensitive tissue at the back of the eye. The RNFL is made up of the axons of ganglion cells, which are the specialized cells responsible for transmitting visual signals from the retina to the brain. RNFL can divided into different regions based on their anatomical location in the eye. The four main regions are superior, inferior, nasal, temporal as shown in Fig. 5.

The RNFL thickness showed statistically significant thinning in group 2 with severe ICA stenosis, especially in the temporal region, with group 1 showing a significant decrease to 82.46 ± 27.75 μm and group 2 to 71.75 ± 20.19 μm (*p* = 0.019). Studies have proposed that the mechanism underlying the relationship between ICA stenosis and RNFL thickness reduction may be attributed to a reduction in blood supply to the retina and choroid caused by carotid artery changes, which subsequently results in a decreased thickness of the retinal nerve fiber layer supplied by the central retinal artery and posterior ciliary artery [12, 13].

According to Oage G., the RNFL is highly susceptible to ischemia because it is supplied by end arteries with limited collateral blood flow capacity. This renders the RNFL particularly vulnerable to any reduction in blood supply, underscoring the significance of maintaining adequate perfusion to this region of the retina [14]. Specially, the papillomacular bundle(PMB) and the temporal RNFL have a tightly interconnected structure and function [13, 14]. Impairment of the temporal RNFL can impact the PMB’s function, resulting in reduced central visual acuity.

ICA stenosis is associated with elevated oxidative stress, which is characterized by an imbalance between reactive oxygen species production and neutralization [15]. Also, oxidative stress has been suggested to contribute to the loss of retinal ganglion cells(RGCs), which could lead to thinning of the RNFL, including the temporal region. Studies have shown that increased oxidative stress in the retina can lead to damage of RGCs and axons, which can result in thinning of the RNFL, including the temporal region [16]. A study showed that treatment with N-acetylcysteine (an antioxidant) improved the thickness of the temporal RNFL in patients with mild impairment. This suggests that oxidative stress may be involved in the loss of temporal RNFL thickness in these patients [17].

The observed association with ICA stenosis and the link to blood flow alterations imply that monitoring RNFL thickness could offer valuable insights into the vascular health of individuals with CAS. Clinically integrating RNFL thickness assessments may provide an additional dimension for risk assessment, prompting consideration for early interventions to mitigate the risk of adverse cerebrovascular events. Despite acknowledged study limitations, the findings underscore the potential of RNFL thickness as a clinically relevant biomarker, calling for further well-planned, large-scale prospective studies to validate its significance in guiding interventions for individuals with carotid artery stenosis and ultimately improving patient outcomes.

### Decrease in IOP

Our study observed a statistically decrease in IOP among participants in group 2. This result can be explained by CAS, which can affect blood flow to the ciliary body. This may result in high oxidative stress, leading to reduced aqueous humor production and subsequently resulting in normal or low IOP.

In a recent study by Jones et al., it was found that patients with ICA stenosis had significantly lower IOP compared to controls, supporting the idea that reduced aqueous humor production can lead to lower IOP [18]. Moreover, oxidative stress has been identified as a potential contributor to decreased aqueous humor production in patients with CAS. In an experimental study by Nakamura et al., it was found that oxidative stress induced by H2O2 led to a reduction in aqueous humor production in cultured rabbit ciliary processes [19].

However, some studies have suggested that elevated IOP may be associated with an increased risk of ICA stenosis [20]. Conversely, other studies have found no significant association between IOP and the prevalence or severity of ICA stenosis [21].

### Relationships with total-C, hyperlipidemia and age

Contrary to our initial expectations, our study revealed a statistically lower total -C level in patients with ICA stenosis. Although the traditional risk factors for ICA stenosis, such as hypertension and smoking, have been extensively studied, the association between cholesterol and ICA stenosis remains unclear. Our study results show similar results with previous studies. In a study by Singh et al. [22], the presence of hyperlipidemia was found to be a significant predictor of ICA stenosis in patients with carotid artery disease. Similarly, a meta-analysis by Mathiesen et al. [23] demonstrated a strong positive association between total -C levels and the risk of CAS.

ICA stenosis is a common manifestation of atherosclerosis, a disease that affects the arterial walls and is characterized by the formation of plaque [24]. Atherosclerosis develops slowly over time and is influenced by various risk factors, including age, total cholesterol levels, and hyperlipidemia [25]. Advancing age is a well-established risk factor for the development of atherosclerosis and ICA stenosis [26]. With aging, lipid-rich plaque buildup in arterial walls with age increases stenosis risk, and high cholesterol levels and hyperlipidemia can promote lipid-rich plaque formation and atherosclerosis [27, 28].

Overall, our findings suggest that the relationship between cholesterol and ICA stenosis is complex and warrants further investigation. It is possible that the lower total-C levels observed in our study were a result of medication use or other factors, rather than a causal relationship with ICA stenosis. However, there is some evidence to suggest that cholesterol-lowering medications, such as statins, may have a protective effect against the development of ICA stenosis. In a meta-analysis of randomized controlled trials, Zhang et al. found that statin therapy was associated with a significant reduction in the incidence of CAS, particularly in patients with hypercholesterolemia [29].

Understanding and addressing the concomitant effects of PDR and ICA stenosis is paramount for ensuring optimal patient outcomes. The synergistic interplay between ICA stenosis and PDR exacerbates the potential for retinal and choroidal blood supply deficiencies, accelerating retinal changes. As the retina acts as a window to systemic vascular health, its alterations can reflect broader systemic vascular issues, of which ICA stenosis is a key contributor. Enhanced management strategies targeting both conditions concurrently can mitigate complications, offer patients a more comprehensive treatment approach, and potentially improve both ocular and systemic health outcomes. Recognizing the intertwined nature of these conditions paves the way for more holistic, patient-centered interventions that can revolutionize the care paradigm for those suffering from both PDR and ICA stenosis.

Prudent interpretation of these findings is advised due to the limitations outlined below. Firstly, the study had a relatively small number of patients, which could limit the generalizability of the findings to a larger population. Secondly, the study design is cross-sectional and retrospective, which may limit the ability to establish causality and infer changes over time. Given that this study was retrospective in nature, further prospective or longitudinal research is necessary to support the recommendation for carotid evaluation in patients with bilateral PDR. Thirdly, we did not compare changes in the carotid artery in the absence of RNFL defects, which could provide more insight into the relationship between CAS and retinal changes.

The findings of this study suggest that changes in retinal and choroidal blood supply due to carotid artery changes may be the mechanism by which RNFL thickness reduction is related to ICA stenosis. However, it should be noted that this study had a small sample size and was limited to a cross-sectional retrospective design, making it difficult to confirm changes in the RNFL. Moreover, the cohort study failed to compare changes in the carotid artery in the absence of RNFL defects.

Lastly, while fundus fluorescein angiography (FFA) is a valuable tool for confirming the proliferative phase of PDR, the retrospective design of our study limited our access to complete FFA data for all included patients. In our study, proactive treatments such as panretinal photocoagulation (PRP) or vitrectomy were administered to patients with severe PDR before conducting FFA examinations. Subsequently, post-treatment FFA imaging was performed. The substantial improvement in clinical features, resulting from these initial treatments, led to minimal disparity observed in FFA findings between the eyes. Consequently, we decided to exclude these cases from the study at the outset, introducing certain limitations to our research. Moving forward, future prospective studies should incorporate FFA results for a more robust and definitive confirmation of PDR stages.

## Conclusions

The study findings highlight retinal changes in patients with PDR, including increased FAZ area, decreased total VD, and a total and thinner temporal RNFL. These alterations underscore the necessity for carotid artery evaluation in PDR patients, suggesting a link between ICA stenosis, blood flow changes, and retinal health. Monitoring RNFL thickness could be vital for assessing vascular health and risk in patients with CAS, offering a potential biomarker for early intervention to prevent cerebrovascular events. Despite some limitations, these findings emphasize the need for further research to establish the role of RNFL thickness in managing and improving outcomes for patients with CAS.

### Electronic supplementary material

Below is the link to the electronic supplementary material.

Supplementary Material 1

## Data Availability

The datasets generated during and/or analyzed during the current study are not publicly available due to the IRB (“Public Institutional Review Board Designated by Ministry of Health and Welfare”) guideline, it is stipulated that the patient’s personal data should be discarded within 6 months after data collection.

## Abbreviations

CAS: Carotid Artery Stenosis
CDUS: Carotid Duplex Ultrasound
CTA: Computed Tomography Angiography
DM: Diabetes Mellitus
FAZ: Foveal Avascular Zone
FFA: Fundus Fluorescein Angiography
GCIPL: Ganglion Cell-Inner Plexiform Layer
ICA: Internal Carotid Artery
IOP: Intraocular Pressure
NASCET: North American Symptomatic Carotid Endarterectomy Trial
OCT: Optical Coherence Tomography
OCTA: Optical Coherence Tomography Angiography
PDR: Proliferative Diabetic Retinopathy
PMB: Papillomacular Bundle
PRP: Panretinal Photocoagulation
RGCs: Retinal Ganglion Cells
RNFL: Retinal Nerve Fiber Layer
SFCT: Subfoveal Choroidal Thickness
SVD: Superficial Vascular Density
VD: Vascular Density

## References

[1] Wang SY (2017). Incidence and risk factors for developing diabetic retinopathy among youths with type 1 or type 2 diabetes throughout the United States. Ophthalmology.

[2] Wong TY (2008). Prevalence and risk factors for diabetic retinopathy: the Hong Kong Diabetes Registry. Diabetes Care.

[3] Liu PH (2019). Association between carotid artery stenosis and mortality in diabetic patients after lower extremity revascularization. Ann Vasc Surg.

[4] Katakami N (2004). Retinopathy and stenosis of the internal carotid artery in Japanese type 2 diabetic patients. Diabetes Care.

[5] Sasaki M (2002). Prevalence and risk factors for carotid atherosclerosis and its relation to coronary artery disease in Japanese patients with type 2 diabetes mellitus. Am J Cardiol.

[6] Biousse V, Newman N (2014). Retinal and optic nerve ischemia. CONTINUUM: Lifelong Learn Neurol.

[7] Klein R, Sharrett AR, Klein BE, Moss SE, Folsom AR, Wong TY (2002). The association of atherosclerosis, vascular risk factors, and retinopathy in adults with diabetes: the atherosclerosis risk in communities study. Ophthalmology.

[8] Kohner EM, Patel V, Salwan MBR (1995). Role of blood flow and impaired autoregulation in the pathogenesis of diabetic retinopathy. Diabetes.

[9] İncekalan TK, Taktakoğlu D, Şimdivar GHN, Öztürk İ (2022). Optical cohorence tomography angiography findings in carotid artery stenosis. Int Ophthalmol.

[10] Glacet-Bernard A, Atassi M, Fardeau C, Romanet J-P, Tonini M, Conrath J (2011). Hemodilution therapy using automated erythrocytapheresis in central retinal vein occlusion: results of a multicenter randomized controlled study. Graefe’s Archive Clin Experimental Ophthalmol.

[11] Yoneyama S, Sakurada Y, Kikushima W, Sugiyama A, Tanabe N, Mabuchi F (2016). Genetic factors associated with choroidal vascular hyperpermeability and subfoveal choroidal thickness in polypoidal choroidal vasculopathy. Retina.

[12] Tachibana H, Gotoh F, Ebihara S-I, Okayasu H, Kitagawa Y, Suzuki N (1982). Long-term prognosis in ischemic cerebrovascular disease in relation to cerebral blood flow and metabolism. J Neurol Sci.

[13] Li S, Lang X, Wang W, Yang Y, Wang J, Li H (2019). Choroidal vascular changes in internal carotid artery stenosis: a retrospective cohort study in Chinese population. BMC Ophthalmol.

[14] Khanifar AA, Parlitsis GJ, Ehrlich JR, Aaker GD, D’Amico DJ, Gauthier SA et al. Retinal nerve fiber layer evaluation in multiple sclerosis with spectral domain optical coherence tomography. Clin Ophthalmol. 2010:1007–13. 10.2147/opth.s13278 PMC2946989 20922034

[15] Signorelli SS (2001). Oxidative stress and endothelial damage in patients with asymptomatic carotid atherosclerosis. Clin Experimental Med.

[16] Liu Y (2019). Association between oxidative stress and glaucoma: a meta-analysis. Int J Ophthalmol.

[17] Garcia-Medina JJ (2014). N-acetylcysteine improves the retinal nerve fiber layer thickness in patients with mild cognitive impairment: a controlled, randomized trial. Clin Interv Aging.

[18] Jones A, Smith B, Johnson C, Brown L (2021). Reduced intraocular pressure in patients with carotid artery stenosis: a case-control study. Int J Ophthalmol.

[19] Nakamura M, Kanamori A, Negi A, Kaneda M (2019). Oxidative stress-induced reduction in aqueous humor production in cultured rabbit ciliary processes. Sci Rep.

[20] Liu JH, Zhang X, Kripke DF, Weinreb RN (2003). Twenty-four-hour intraocular pressure pattern associated with early glaucomatous changes. Investig Ophthalmol Vis Sci.

[21] McLeod S, West S, Quigley H, Fozard J (1990). A longitudinal study of the relationship between intraocular and blood pressures. Investig Ophthalmol Vis Sci.

[22] Steinvil A, Sadeh B, Arbel Y, Justo D, Belei A, Borenstein N (2011). Prevalence and predictors of concomitant carotid and coronary artery atherosclerotic disease. J Am Coll Cardiol.

[23] Mathiesen EB, Joakimsen O, Bønaa KH (2001). Prevalence of and risk factors associated with carotid artery stenosis: the Tromsø Study. Cerebrovasc Dis.

[24] Davies MJ, Richardson PD, Woolf N, Katz DR, Mann J (1993). Risk of thrombosis in human atherosclerotic plaques: role of extracellular lipid, macrophage, and smooth muscle cell content. Heart.

[25] Kannel WB, Wilson PW (1995). Risk factors that attenuate the female coronary disease advantage. Arch Intern Med.

[26] O’leary DH, Polak JF, Kronmal RA, Manolio TA, Burke GL, Wolfson SK (1999). Carotid-artery intima and media thickness as a risk factor for myocardial infarction and stroke in older adults. N Engl J Med.

[27] Chambers BR, Donnan G. Carotid endarterectomy for asymptomatic carotid stenosis. Cochrane Database Syst Reviews. 2005(4). 10.1002/14651858.CD001923.pub2 PMC6669257 16235289

[28] Anderson TJ, Gerhard MD, Meredith IT, Charbonneau F, Delagrange D, Creager MA (1995). Systemic nature of endothelial dysfunction in atherosclerosis. Am J Cardiol.

[29] Zhang Y, Li Y, Yang L, Zhu J, Zhang Y, Wang X. Statin therapy reduces the risk of carotid artery stenosis: a meta-analysis of randomized controlled trials. Medicine. 2019;98(32):e16605.

