# Supplementary material for: Risk factors of internal carotid artery stenosis in patients with proliferative diabetic retinopathy: an analysis using optical coherence tomography and optical coherence tomography angiography
Source: BMC Ophthalmol. 2024 Apr 9;24:156. doi: 10.1186/s12886-024-03391-z (PMC11003116; doi:10.1186/s12886-024-03391-z)
Supplement: Supplementary file 1 — Supplementary Material 1 [file 12886_2024_3391_MOESM1_ESM.pptx]

## Slide 1
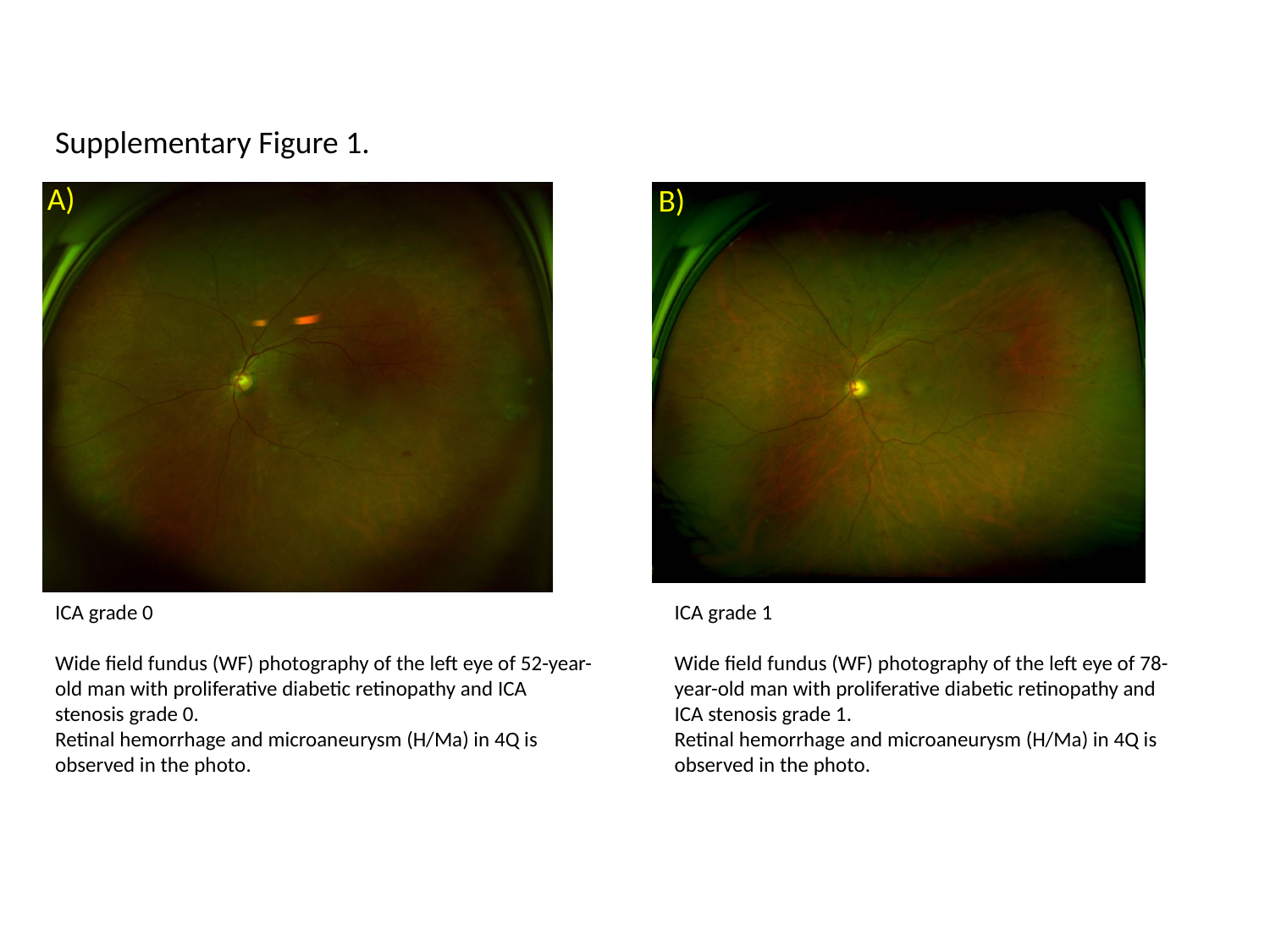

Supplementary Figure 1.
A)
B)
ICA grade 0
Wide field fundus (WF) photography of the left eye of 52-year-old man with proliferative diabetic retinopathy and ICA stenosis grade 0.
Retinal hemorrhage and microaneurysm (H/Ma) in 4Q is observed in the photo.
ICA grade 1
Wide field fundus (WF) photography of the left eye of 78-year-old man with proliferative diabetic retinopathy and ICA stenosis grade 1.
Retinal hemorrhage and microaneurysm (H/Ma) in 4Q is observed in the photo.

## Slide 2
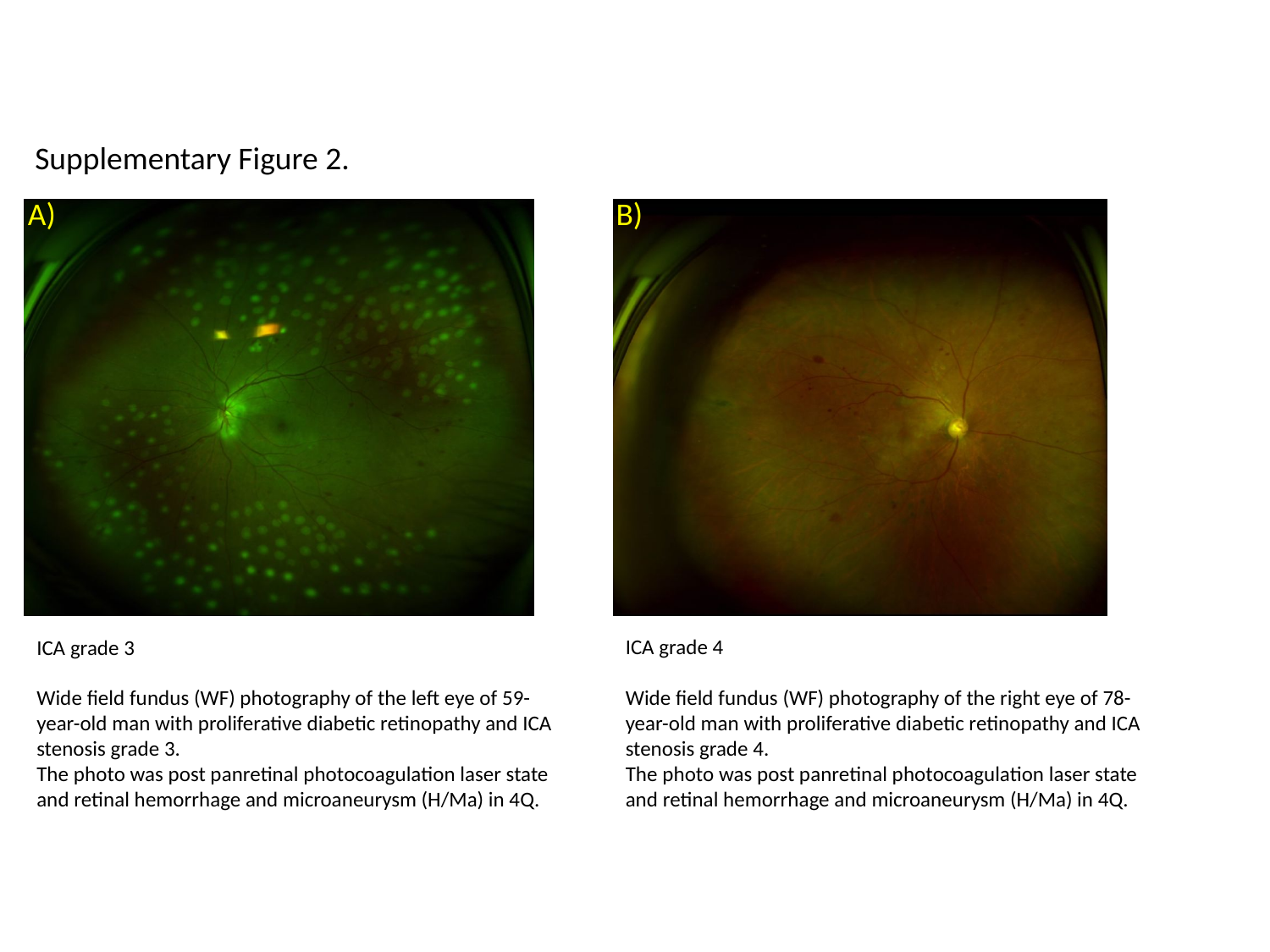

Supplementary Figure 2.
B)
A)
ICA grade 3
Wide field fundus (WF) photography of the left eye of 59-year-old man with proliferative diabetic retinopathy and ICA stenosis grade 3.
The photo was post panretinal photocoagulation laser state and retinal hemorrhage and microaneurysm (H/Ma) in 4Q.
ICA grade 4
Wide field fundus (WF) photography of the right eye of 78-year-old man with proliferative diabetic retinopathy and ICA stenosis grade 4.
The photo was post panretinal photocoagulation laser state and retinal hemorrhage and microaneurysm (H/Ma) in 4Q.

## Slide 3
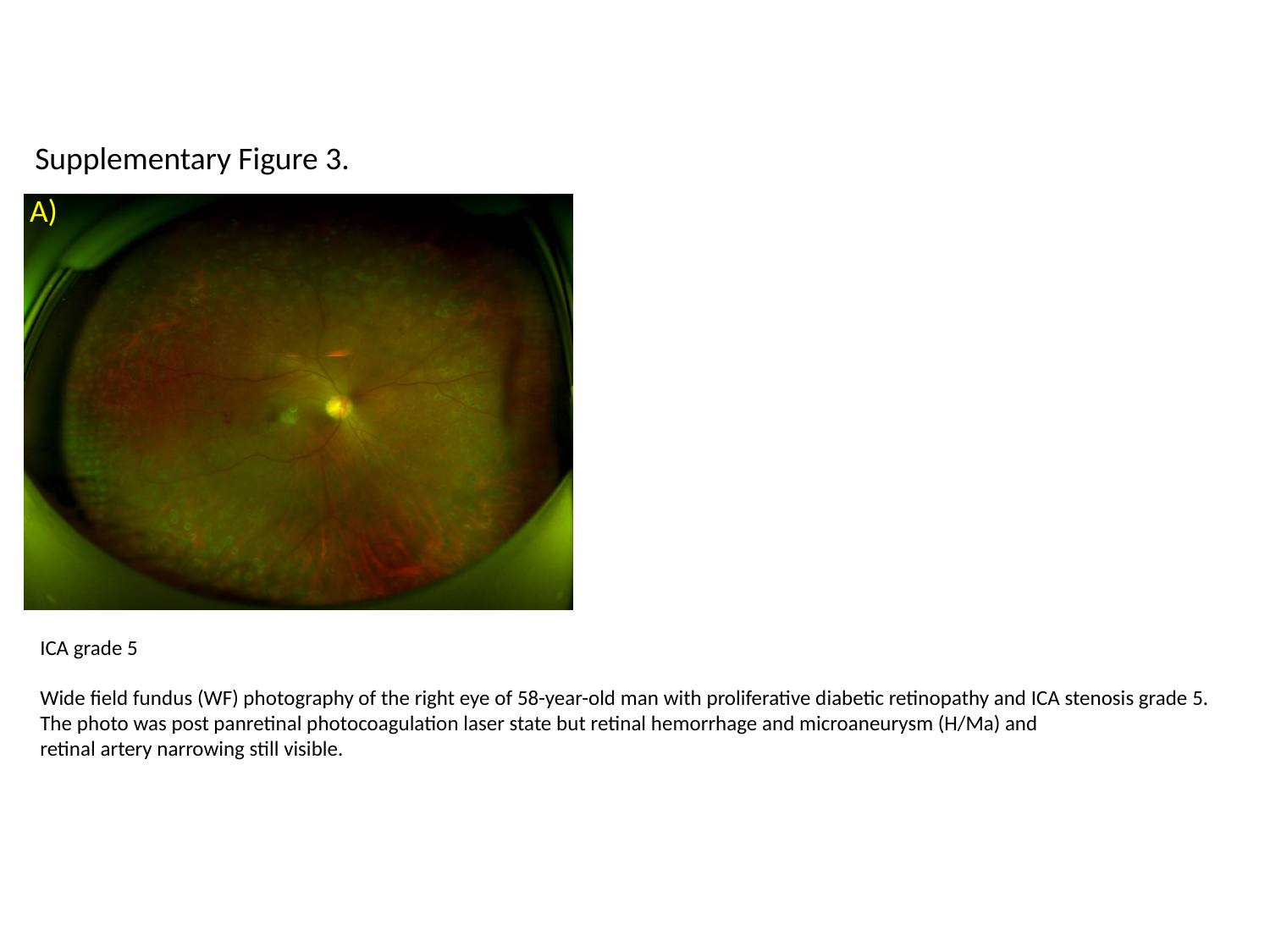

Supplementary Figure 3.
A)
ICA grade 5
Wide field fundus (WF) photography of the right eye of 58-year-old man with proliferative diabetic retinopathy and ICA stenosis grade 5.
The photo was post panretinal photocoagulation laser state but retinal hemorrhage and microaneurysm (H/Ma) and
retinal artery narrowing still visible.

## Slide 4
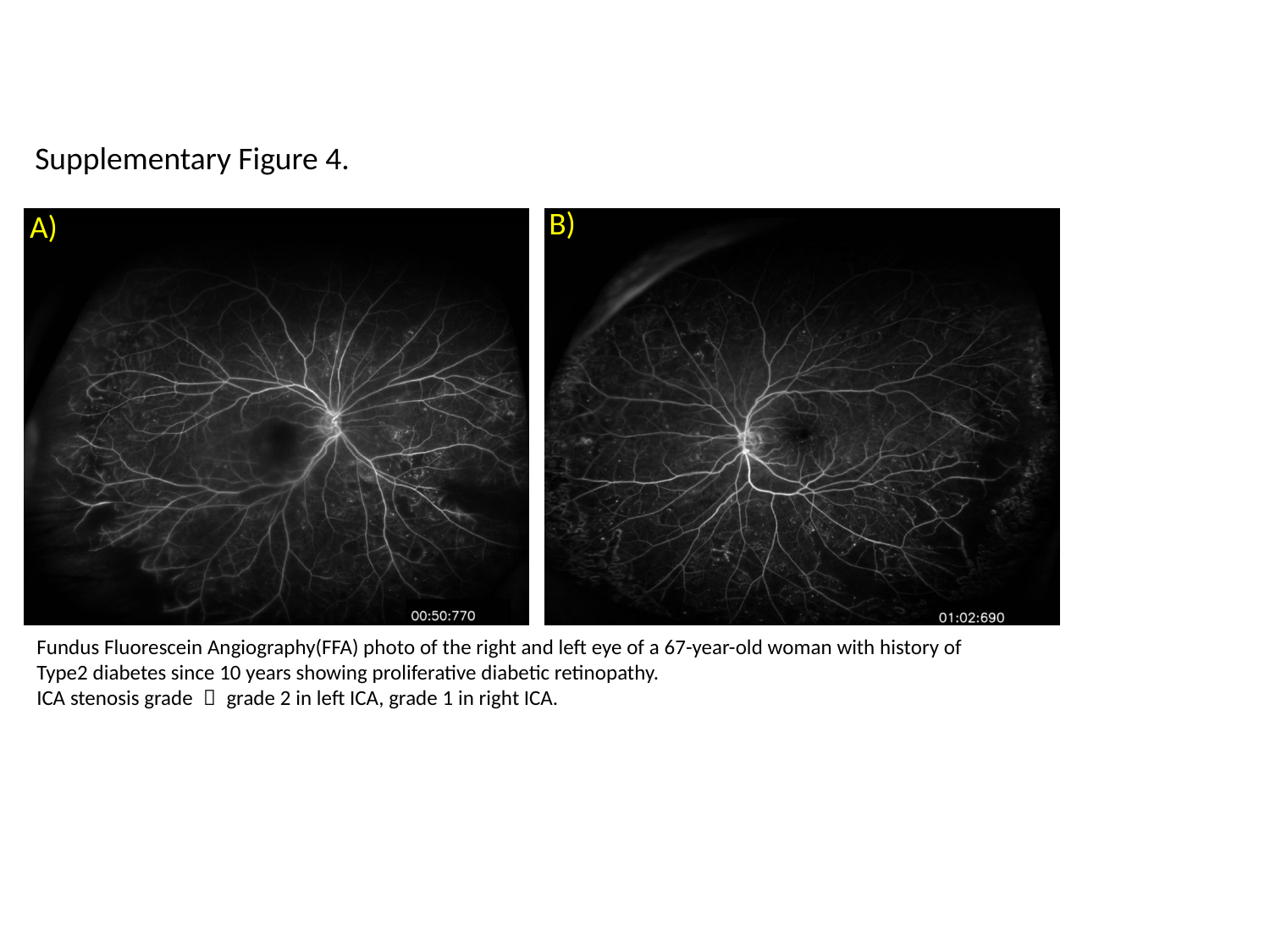

Supplementary Figure 4.
B)
A)
Fundus Fluorescein Angiography(FFA) photo of the right and left eye of a 67-year-old woman with history of Type2 diabetes since 10 years showing proliferative diabetic retinopathy.
ICA stenosis grade 는 grade 2 in left ICA, grade 1 in right ICA.
